# Interferon Regulatory Factor-1 Protects from Fatal Neurotropic Infection with Vesicular Stomatitis Virus by Specific Inhibition of Viral Replication in Neurons

**DOI:** 10.1371/journal.ppat.1003999

**Published:** 2014-03-27

**Authors:** Sharmila Nair, Kristin Michaelsen-Preusse, Katja Finsterbusch, Sabine Stegemann-Koniszewski, Dunja Bruder, Martina Grashoff, Martin Korte, Mario Köster, Ulrich Kalinke, Hansjörg Hauser, Andrea Kröger

**Affiliations:** 1 Research Group Innate Immunity and Infection, Helmholtz Centre for Infection Research, Braunschweig, Germany; 2 Department of Cellular Neurobiology, Technical University Braunschweig, Braunschweig, Germany; 3 Immune Regulation Group, Helmholtz Centre for Infection Research, Braunschweig, Germany; 4 Infection Immunology Group, Department of Medical Microbiology, Otto-von-Guericke-University Magdeburg, Magdeburg, Germany; 5 Research Group Neuroinflammation and Neurodegeneration, Helmholtz Centre for Infection Research, Braunschweig, Germany; 6 Department of Gene Regulation and Differentiation, Helmholtz Centre for Infection Research, Braunschweig, Germany; 7 Institute for Experimental Infection Research, TWINCORE, Hannover, Germany; Kantonal Hospital St. Gallen, Switzerland

## Abstract

The innate immune system protects cells against invading viral pathogens by the auto- and paracrine action of type I interferon (IFN). In addition, the interferon regulatory factor (IRF)-1 can induce alternative intrinsic antiviral responses. Although both, type I IFN and IRF-1 mediate their antiviral action by inducing overlapping subsets of IFN stimulated genes, the functional role of this alternative antiviral action of IRF-1 in context of viral infections *in vivo* remains unknown. Here, we report that IRF-1 is essential to counteract the neuropathology of vesicular stomatitis virus (VSV). IFN- and IRF-1-dependent antiviral responses act sequentially to create a layered antiviral protection program against VSV infections. Upon intranasal infection, VSV is cleared in the presence or absence of IRF-1 in peripheral organs, but IRF-1^−/−^ mice continue to propagate the virus in the brain and succumb. Although rapid IFN induction leads to a decline in VSV titers early on, viral replication is re-enforced in the brains of IRF-1^−/−^ mice. While IFN provides short-term protection, IRF-1 is induced with delayed kinetics and controls viral replication at later stages of infection. IRF-1 has no influence on viral entry but inhibits viral replication in neurons and viral spread through the CNS, which leads to fatal inflammatory responses in the CNS. These data support a temporal, non-redundant antiviral function of type I IFN and IRF-1, the latter playing a crucial role in late time points of VSV infection in the brain.

## Author Summary

IRFs are a family of transcription factors that play a key role in viral defense. Apart from their function in the adaptive immune system, recent work revealed that several IRFs contribute to antiviral response independent of secreted IFN. IRFs have been developed earlier in evolution than IFN and are regarded as precursor of today's IFN system, acting only on an intrinsic level. IRF-1 by itself exhibits antiviral effects that are exerted by the induction of a set of genes that overlaps the set of IFN-induced genes (ISGs). Our data show that IRF-1 contributes decisively for the protection of mice from neurotropic Vesicular stomatitis virus (VSV), a virus similar to rabies virus. Mice, deficient in IRF-1, are highly vulnerable to VSV infection and succumb with signs of encephalitis. Although type I IFN action is a prerequisite for survival from the infection, IRF-1 becomes increasingly crucial in neuronal tissue at a time point where clearance of the virus has not been achieved. The data highlight the importance of IRF-1 as an antiviral agent that acts in combination with the IFN system.

## Introduction

The rapid production of type I Interferon (IFN) in response to a viral attack serves as a crucial antiviral defense mechanism in mammals [1]–[3]. The type I IFN response to invading pathogens is a biphasic phenomenon. First, the detection of RNA viruses occurs through the recognition of pathogen associated molecular patterns (PAMPs) by pathogen recognition receptors (PRRs) such as the toll like receptors (TLRs) or retinoic acid inducible gene-1 (RIG-I) like receptors (RLRs) which initiate a signaling cascade to activate transcription factors like interferon regulatory factor (IRF)-3 and NF-κB to induce the type I IFNs [4]–[6]. Second, the IFNs act in an autocrine and paracrine manner to induce IFN stimulated genes (ISGs), the products of which act collectively to interfere with viral replication and spread.

Viruses have developed a broad range of strategies to evade from this powerful arm of innate defense. These strategies involve preventing initial detection by either interfering with the TLR or RLR signaling adaptor proteins such as TRIF or MAVS [7]–[10], by targeting the PRR expression levels [11]–[13] or by even concealing their genetic material to avoid detection [14], [15]. In addition, the viruses express proteins that inhibit IFN action on different levels [16]–[18]. In turn, host cells have developed strategies to overcome these viral evasion mechanisms by adding alternative mechanisms that are not simultaneously declined by the virus.

Recent studies have implicated several IFN-independent antiviral mechanisms which bypass viral inhibition of IFN induction and function. Several IRFs have been shown to drive antiviral defense mechanisms independent of the type I IFN signaling *in vitro*. IRF-3 has been shown to induce a subset of ISGs by direct binding to their promoters before IFN itself can be produced [19], [20]. In addition, IRF-1-dependent but IFN-independent inductions of antiviral effector functions against different viruses have been shown [21], [22]. While mitochondrial MAVS is shown to carry out IFN-dependent antiviral mechanisms, peroxisomal MAVS has been shown to induce an immediate albeit transient induction of anti-viral defense factors by IFN-independent mechanisms [23]. Further proof of concept included a study which showed IRF-1 mediated induction of several ISGs in STAT1^−/−^ cells [24]. IRF-1 and IRF-5 have also been shown to mediate antiviral responses against the hepatitis C virus by an IFN-independent mechanism [25]. In addition to the IRF mediated antiviral mechanisms, some ISGs can be directly induced by viral infection in the absence of IFN production [26].

The transcription factor IRF-1 has been defined as tumor suppressor gene that inhibits cell proliferation enhances apoptosis and controls tumor growth [27]–[29]. IRF-1 also regulates adaptive immune responses by regulation of MHC class I expression and influencing development of NK and T cells [30], [31]. Originally IRF-1 was identified by its binding to DNA sequences, termed IRF-Es, which are common to the promoters of the IFNα/β genes [32], [33]. Thus, they were proposed to be the regulators of type I IFN and IRF-1 was reported to bind to the IFN-stimulated regulatory elements (ISREs) found in many IFN-inducible gene promoters [21], [34], [35].

Vesicular stomatitis virus (VSV) is a cytopathic virus belonging to the same family as the rabies virus and is capable of causing encephalitis in mice. Type I IFN is rapidly induced upon infection and is crucial to prevent death [36]. In fibroblast cells, type I IFN production and action is efficiently blocked by the virus [37] and antiviral response could be overtaken by IRF-1 mediated induction of ISGs [22], [23].

In this study we aimed to uncover the role of IRF-1 mediated antiviral responses by innate immune mechanisms *in vivo*. We found that mice succumbed from VSV infection in the absence of IRF-1 only after neurotropic infections. IRF-1 was essential for survival but needed neither the type I IFN production nor the adaptive immune response for its antiviral effects. A detailed analysis of this system reveals a role of IRF-1 that can be clearly separated from the action of IFN. IRF-1 expression in neurons prevents reoccurrence of viral replication in the brain which cause fatal encephalitis and death of the mice.

## Results

### IRF-1 protects mice from lethal intranasal VSV infection

**Figure 1:**
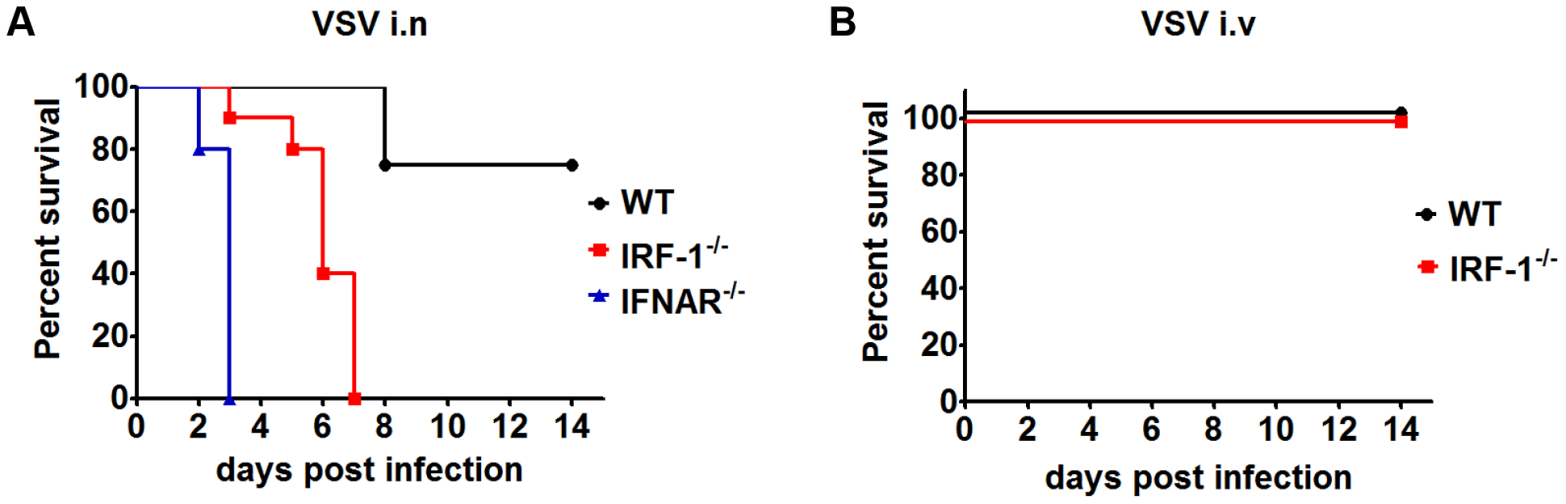
IRF-1 protects mice from lethal intranasal VSV infection. A, Survival analysis of WT (n = 12), IRF-1^−/−^ (n = 10) and IFNAR^−/−^ (n = 5) mice. 8-10-week-old mice were infected intranasally with 5×10^6^ (WT, IRF-1^−/−^) or 10^2^ (IFNAR^−/−^) pfu VSV and mortality was observed for 15 days. B, Survival curves of WT (n = 5) and IRF-1^−/−^ (n = 6) mice after intravenous infection with 5×10^6^ pfu VSV. Data are representative of at least two independent experiments. Survival differences were tested for statistical significance by the log-rank test.

We previously demonstrated that IRF-1 mediates a specific antiviral response *in vitro*, which proves to be essential when viruses evade innate immune responses by antagonizing the induction and function of the type I IFN system [22]. To uncover the functional role of IRF-1 mediated antiviral responses *in vivo*, we used gene knockout mice to study their increased susceptibility to VSV, a neurotropic virus of the *Rhabdoviridae* family that efficiently blocks type I IFN induction and function *in vitro* [38]. We infected WT and IRF-1^−/−^ mice intranasally with 5×10^6^ plaque forming units (pfu) of VSV (Figure 1A). IRF-1^−/−^ mice were found to be susceptible to VSV infections, displaying 100% mortality and died within 7 days after suffering from symptoms of lethargy and weight loss, compared to WT mice showing 20% mortality. IFNAR^−/−^ mice were also employed in the study and were infected with a low dose of 10^2^ pfu of VSV. All IFNAR^−/−^ mice died within 3 days. These data confirm the crucial role of type I IFN in VSV infection. Importantly, IRF-1 is also pivotal for survival of VSV infection, albeit at higher infectious doses.

Upon intranasal infection VSV can enter the CNS by directly infecting the olfactory receptor neurons [39]. To determine whether IRF-1 mediates its antiviral response by a systemic mechanism or by a specific effect in the CNS, we infected IRF-1^−/−^ mice intravenously with VSV (Figure 1B). IRF-1^−/−^ mice showed no increased susceptibility in comparison to WT mice. Interestingly, intranasal infection of mice infected with Influenza A virus PR8/A/34 virus had little impact on the survival of IRF-1^−/−^ mice (Figure S1), indicating a specific role of IRF-1 in neurotropic VSV infections.

To further prove whether IRF-1 is involved in the protective antiviral response of type I IFN in neurotropic VSV infections, we made use of conditional IFNAR^fl/fl^NesCre^+/−^ mice that suffer from the IFNAR deletion specifically in neuroectodermal cells of the CNS [40]. These mice were infected intranasally with VSV (Figure S1). While all IRF-1^+/−^IFNAR^fl/fl^NesCre^+/−^ mice died 6 days post infection, IRF-1 deficient IRF-1^−/−^IFNAR^fl/fl^NesCre^+/−^ mice died between day 2 and 5 post infection. This indicates that apart from the IFN system, IRF-1 is able to execute additional antiviral functions in the brain. Together, these data suggest a pivotal antiviral function of IRF-1 in the brain.

### Impact of IRF-1 on type I IFN levels

VSV replication is strictly controlled by Type I IFN [36], [41]. Since IRF-1 has previously been described to regulate the transcription of IFN-β as well as IFN inducible genes *in vitro* [32], we determined the impact of IRF-1 on the IFN response upon VSV infection. To this end, we took advantage of a Mx2Luc reporter mouse which allows whole body *in vivo* imaging of type I and III response using firefly luciferase as a reporter [42].

**Figure 2:**
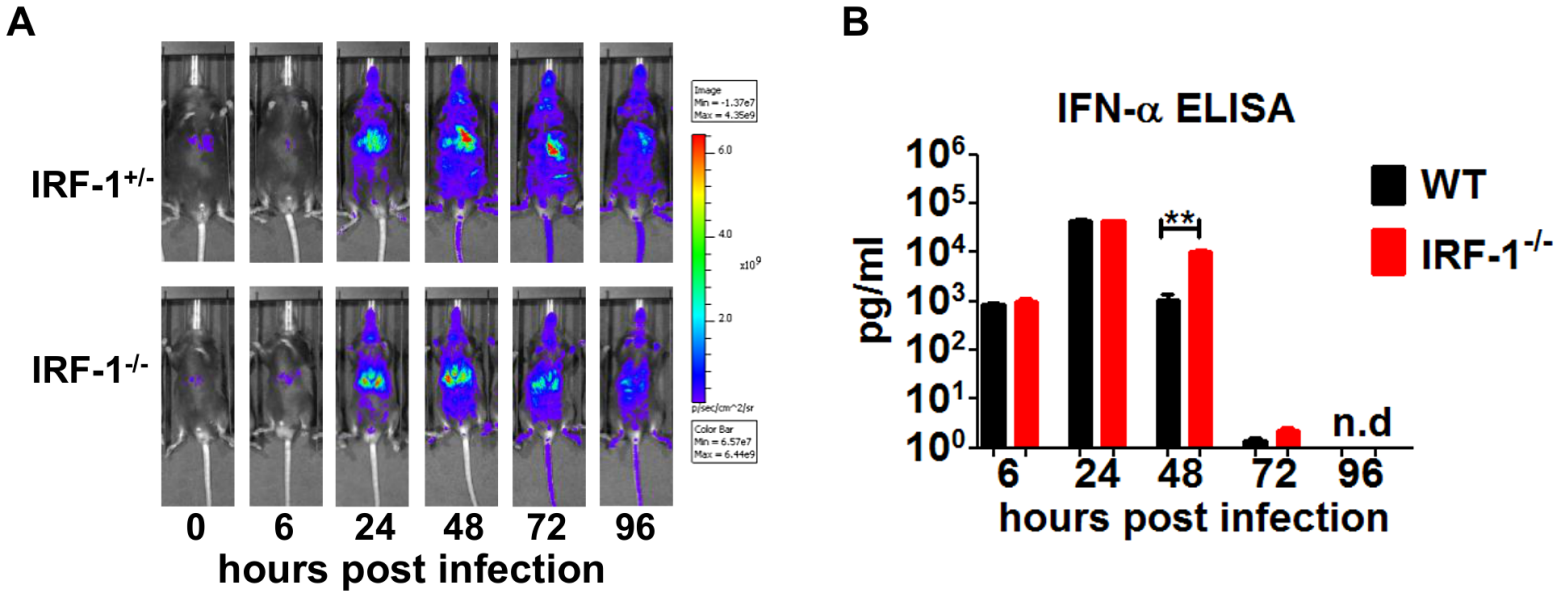
IRF-1 is dispensable for type I IFN response in VSV infections. Mice were infected intranasally with 5×10^6^ pfu of VSV. A, IRF-1^+/−^Mx2Luc and IRF-1^−/−^Mx2Luc transgenic mice were subjected to whole body imaging. Mice were imaged before treatment (0 h) and over time as indicated. Images from a representative mouse are shown. The rainbow scale indicates the number of photons measured per second per cm^2^ per steradian (sr). B, Serum was collected from WT and IRF-1^−/−^ mice at different time points post infection. IFN-α protein level was determined by ELISA. Data represents mean with SEM of 5–6 mice in each group per time point. Asterisks indicate statistical significance calculated by Mann-Whitney test, ** p<0.005

Intranasal VSV infection of IRF-1^+/−^Mx2Luc induced reporter gene expression in the whole body, with a major response in the region of the liver 24 hours post infection (Figure 2A). The signal reached its maximum, 48 hours post infection after which it declined. No differences of luciferase signals was detectable in IRF-1^+/+^Mx2Luc mice (data not shown), IRF-1^+/−^Mx2Luc and IRF-1^−/−^ Mx2Luc mice. *In vivo* imaging of luciferase activity is influenced by the position of the organ, since the emitted light is absorbed by surrounding tissue. Therefore, we performed a detailed analysis of luciferase expression in homogenates of different organs. Our data reveal a slightly higher type I IFN response in some organs of IRF-1^−/−^ mice compared to WT controls (Figure S2). In addition, luciferase signals were also induced in the brains of WT and IRF-1^−/−^ mice. In WT mice the IFN responses in the brains were back to basal levels by day 9 post infection. These results confirmed that deletion of the IRF-1 gene does not lead to diminished levels of type I IFN. We also determined IFN-α serum levels in VSV infected mice (Figure 2B). Equal levels of IFN-α were detectable in the serum of both WT and IRF-1^−/−^ mice at 6 hours and 24 hours post infection. However, IRF-1^−/−^ mice displayed higher IFN-α in the serum 2 days post infection. No IFN-α was detectable in WT and IRF-1^−/−^ mice from day 4 post infection. This corroborates the evidence that there is no deficiency in IFN action and this is not responsible for the increased susceptibility of IRF-1^−/−^ mice.

### IRF-1 mediated antiviral response is independent of the adaptive immune responses, but depends on non-hematopoietic cells

**Figure 3:**
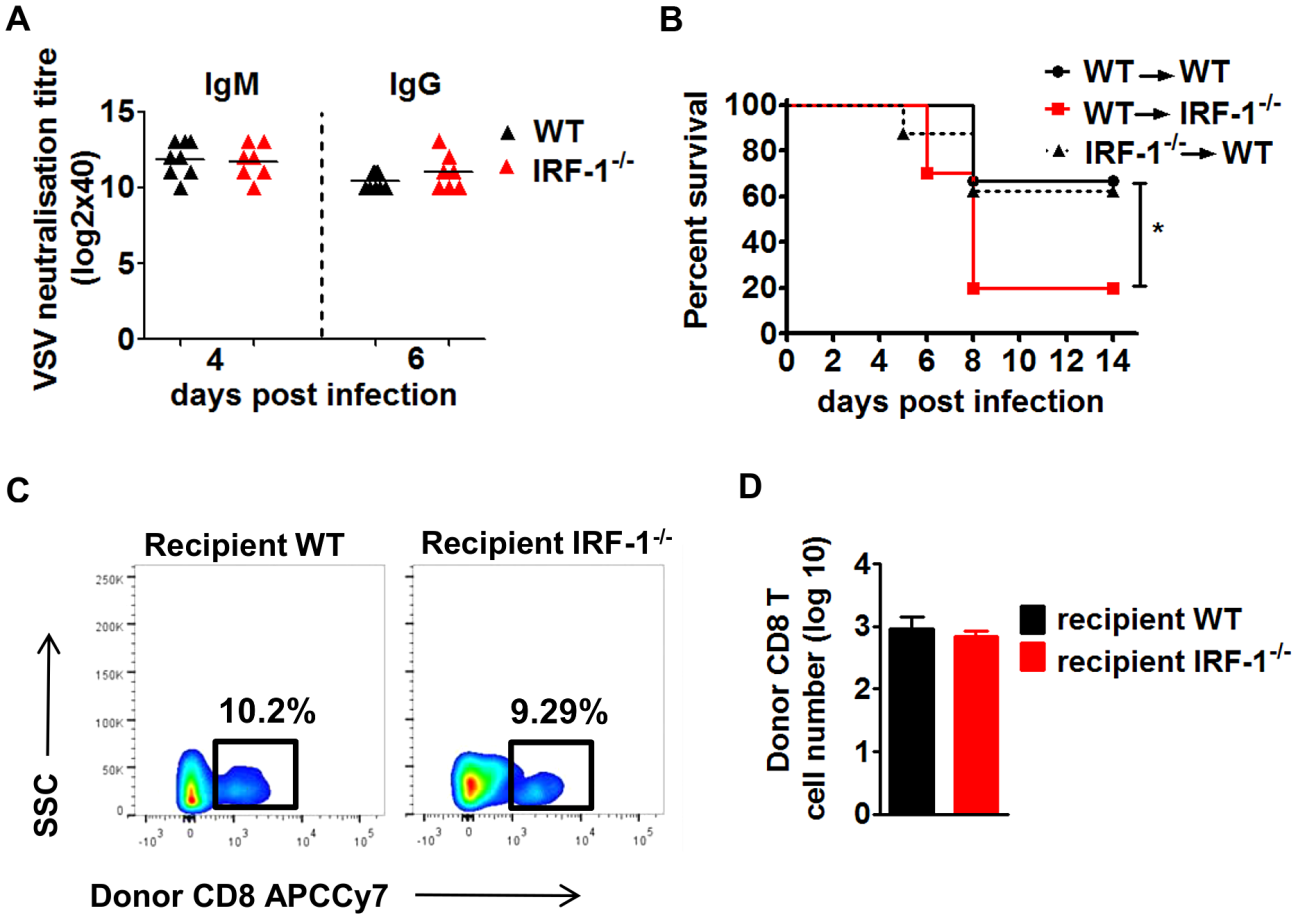
The anti-viral effects of IRF-1 are neither driven by the adaptive immune responses nor by the hematopoietic cells. A, WT and IRF-1^−/−^ mice were infected intranasally with 5×10^6^ pfu VSV and serum samples were collected at the indicated time points. IgM and IgG titers were quantitated by the virus neutralization assay. B/C/D, WT and IRF-1^−/−^ mice were lethally irradiated and reconstituted with bone marrow from IRF-1^−/−^ or WT mice, respectively. After 6–8 weeks, chimeric mice were infected intranasally with 5×10^6^ pfu VSV. B, Survival was monitored and plotted as Kaplan-Meier curves (n = 8–10). Data are representative of two independent experiments. Survival differences were tested for statistical significance by the log-rank test, * p<0.05. C,D Leukocytes were isolated from brains of chimeric mice (n = 6–9) 6 days post infection, stained for CD45.1 and CD8, and analyzed by flow cytometry, C, Representative flow cytometry profiles of WT CD8^+^ T cells from donor hematopoietic cells in the recipient irradiated WT and IRF-1^−/−^ mice. D, Total cell numbers for infiltrating donor WT CD8+ T cells in the brains of recipient WT and IRF-1^−/−^ mice.

Earlier work has demonstrated the importance of the adaptive immune system for survival during VSV infection. A depressed neutralizing antibody response in the periphery promotes early dissemination of the virus to the CNS [37]. We evaluated whether IRF-1 modulates the humoral immune response by determining VSV neutralizing antibodies (Figure 3A). Similar levels of VSV specific IgM and IgG titers were detected in IRF-1^−/−^ and WT mice. These data suggest that increased susceptibility of IRF-1^−/−^ mice to VSV infection is not due to a defect in the B cell response.

While the systemic IFN response and neutralizing antibodies do not impact the IRF-1 mediated antiviral responses, we asked if the antiviral action of IRF-1 is due to stromal and brain resident cells or whether the hematopoietic system facilitates an indirect control of VSV infection. Since the hematopoietic cells are sensitive to irradiation while the brain resident cells are resistant [40], we established reciprocal bone marrow chimeric mice. These bone marrow chimeric mice were infected with VSV (Figure 3B). IRF-1^−/−^ → WT chimeras were capable of limiting infection and showed comparable susceptibility to WT → WT mice, suggesting that IRF-1 expression in WT host stromal and resident brain cells is critical to control the virus. This is supported by inverse experiments in which WT → IRF-1^−/−^ chimeras were unable to rescue the IRF-1^−/−^ mice. Analysis of brain infiltrating T cells in the chimeric mice revealed no differences in the number of donor CD8^+^ T cells (Figure 3C/D). Moreover, CD8^+^ T cells in the brains of IRF-1^−/−^ or WT recipients were neither activated nor antigen specific (data not shown). Thus, the IRF-1 mediated antiviral effects are driven primarily by radiation-resistant stromal or brain resident cells.

### Dynamics of viral replication in the brain of wild-type and IRF-1^−/−^ mice

**Figure 4:**
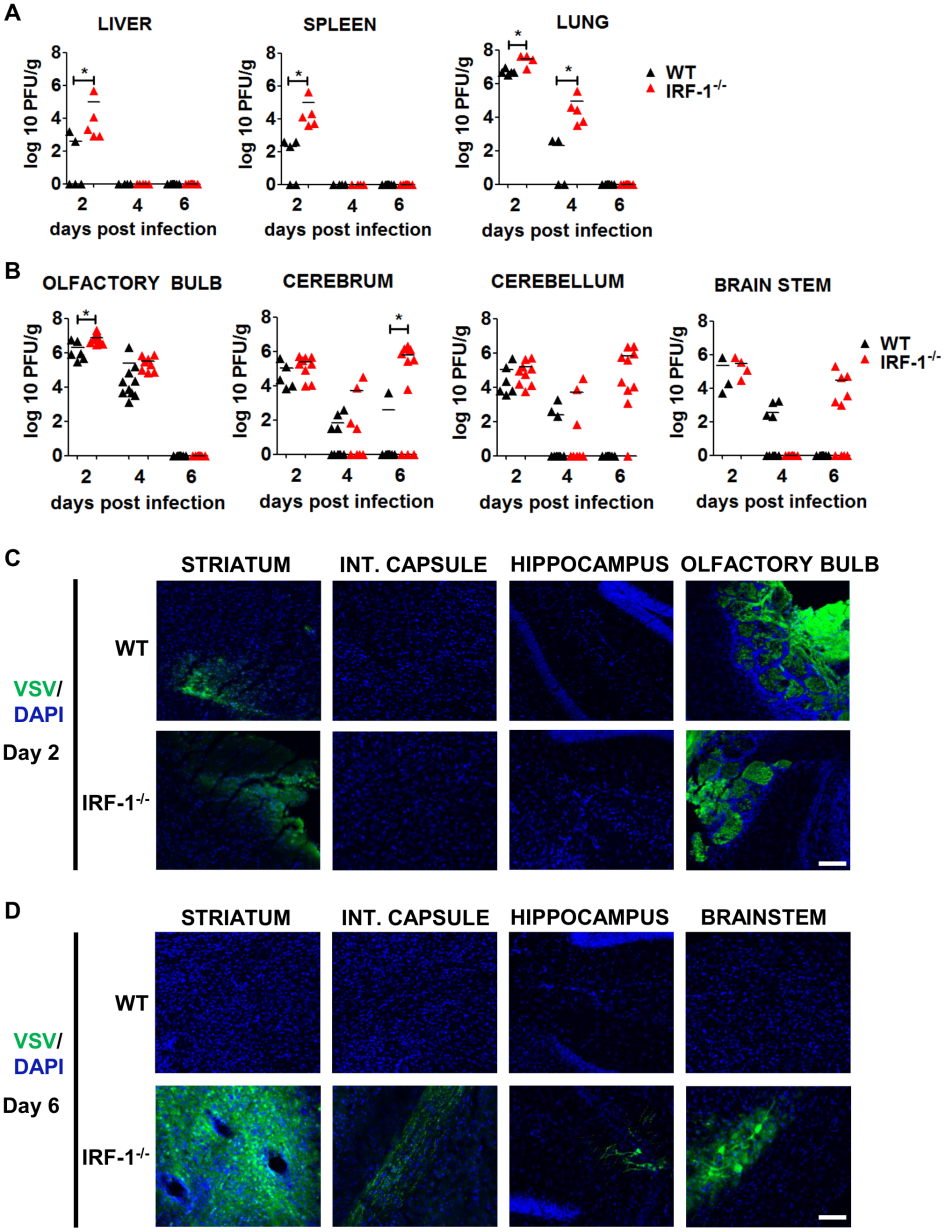
IRF-1 mediated antiviral effect is critical for viral replication during later stages of viral replication in the brain. WT and IRF-1^−/−^ mice infected intranasally with 5×10^6^ pfu of VSV or VSV-eGFP and viral burden was quantitated by plaque assay from A, peripheral organs- liver, spleen, lung and B, brain parts- olfactory bulb, cerebrum, cerebellum and brain stem. Data are representative of at least two independent experiments. Asterisks indicate statistical significance calculated by Mann-Whitney test, * p<0.05. C/D, Representative pictures of immunohistological analysis of VSV-eGFP protein in the brain stem and different parts of the cerebrum in IRF-1^−/−^ mice (VSV-eGFP (green), DAPI (blue). C, 2 days post infection, D, 6 days post infection. Scale bar 100 μm

Intranasal administration of a high viral dose of VSV leads to viral replication in the brain as well as in peripheral organs [36]. We therefore determined viral load from various tissues by plaque assay 2, 4, 6 days post intranasal infection with 5×10^6^ pfu of VSV (Figure 4). Viral burden from the peripheral organs i.e. liver, spleen and lungs was higher in IRF-1^−/−^ mice in comparison to WT mice 2 days post infection (Figure 4A). However, viral clearance from the liver and spleen was observed by day 4 and the lung by day 6 in both the WT and IRF-1^−/−^ mice. These data indicate that IRF-1 had an impact in limiting viral replication but clearance of the virus from peripheral organs could be achieved even in the absence of IRF-1.

Intranasal administration leads VSV to spread from the nasal cavity to the olfactory bulb and into the CNS [39], [43]. To examine the impact of IRF-1 during brain infection more carefully, we dissected the mouse brain into four parts, i.e. olfactory bulb, cerebrum, cerebellum and brain stem post infection and determined their viral load by plaque assay at different time points (Figure 4B). Viral titers were detectable in all brain parts of both, WT and IRF-1^−/−^ mice 2 days post infection. WT mice cleared the virus within 6 days. This was also found for the olfactory bulb of IRF-1^−/−^ mice, which showed viral clearance with similar kinetics compared to the peripheral tissues. Interestingly, although viral titers had decreased from all brain parts by day 4 in both WT and IRF-1^−/−^ mice, a surge of viral load was found in the brain tissues of IRF-1^−/−^ mice. High viral titers developed at this time point in the cerebrum, cerebellum and brain stem, suggesting this as a cause for the death of these animals. To investigate the impact of IRF-1 on viral replication and spread we performed immunohistological analysis from different parts of the cerebrum and the brain stem (Figure 4C/D). Analysis of the brain 2 days post infection revealed, that at this time point, only the olfactory bulb and the striatum of the cerebrum is infected by VSV (Figure 4C). By day 6, VSV-eGFP could be detected in neurons of the brain stem, striatum, internal capsule and hippocampus in IRF-1^−/−^. In contrast, no virus was detectable in brains of infected WT mice at this time point (Figure S3). Interestingly, high VSV-eGFP was detectable in axonal fibers of the internal capsule, which connects the cerebral cortex and the pyramids of the medulla. This indicates that in the absence of IRF-1, VSV is able to spread to different regions within the cerebrum and other parts of the brain. These data also indicate that IRF-1 plays a minor role during the early phase of viral brain infection and clearance. However, IRF-1 is crucial in the control of a second phase of viral replication in the cerebrum, cerebellum, and brain stem. Furthermore, IRF-1 is important for restricting viral spread.

### IRF-1 mediated antiviral activity presents a secondary wave of virus propagation that is not controlled by IFN

**Figure 5:**
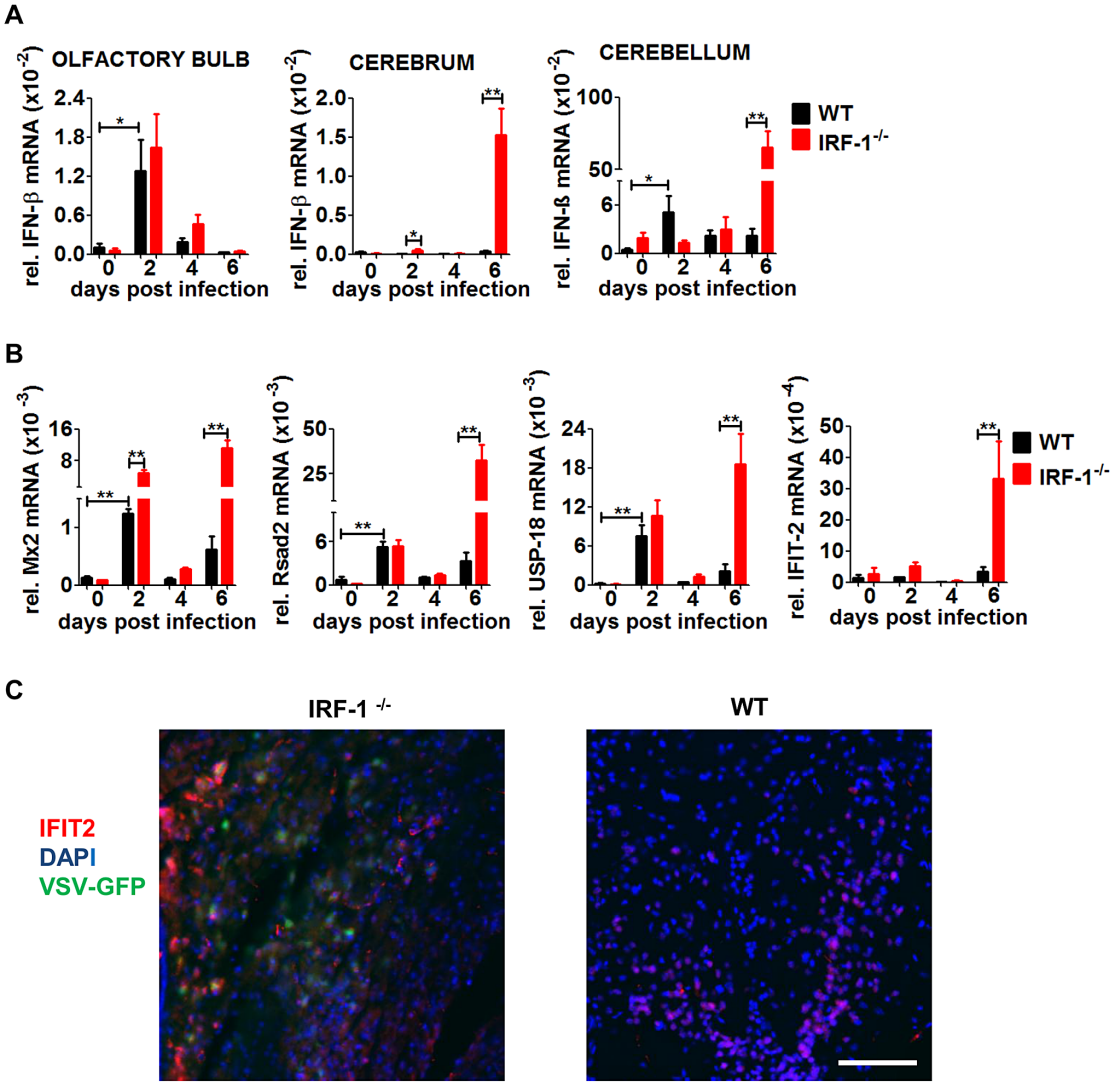
IFN-β and ISG mRNA expression in neurotropic VSV infection. Mice were infected intranasally with 5×10^6^ pfu of VSV or VSV-eGFP. A, Expression level of IFN-β in different brain parts was determined by real-time RT-PCR, A, IFN-β. B, Expression level of Mx2, Rsad2 USP-18 and IFIT-2 in the cerebrum was determined by real-time RT-PCR. Data represents mean with SEM of 3–7 mice in each group per time point. Asterisks indicate statistical significance calculated by Mann-Whitney test, ** p<0.005, * p<0.05. C, Representative pictures of immunohistological analysis of VSV-eGFP and IFIT-2 protein 6 days post infection in the cerebrum of IRF-1^−/−^ and WT mice. IFIT2 (red), DAPI (blue), VSV-eGFP (green). Scale bar is 50 μm.

To dissect the different roles of type I IFN and IRF-1 in the brain during VSV infection, we determined IFN-β mRNA in the brain parts after intranasal infection with VSV (Figure 5A). Interestingly, the IFN-β mRNA expression pattern and kinetics differed within the different brain regions. In the olfactory bulb IFN-β expression was detectable 2 days post infection, and its expression level declined to basal levels by day 4. No difference in IFN-β mRNA expression level was detectable between WT and IRF-1^−/−^ mice. These data correlate to the kinetics of viral load where IRF-1 has no impact in the control of viral replication in the olfactory bulb. In the cerebrum and cerebellum of IRF-1^−/−^ mice, we observed a resurgence of IFN-β mRNA induction at day 6 post infection, which correlated to higher viral replication in the absence of IRF-1.

The kinetics of IFN-β expression levels within the cerebrum and cerebellum indicated little if any IFN-β mRNA was produced at day 2 post infection. However, significant amounts of IFN-β were found only in the cerebrum and cerebellum of IRF-1^−/−^ mice, 6 days post infection. These data indicate that loss of IRF-1 leads to higher viral replication in the late phase of virus infection, although IFN-β is expressed.

This corresponds well to the kinetic of enhanced viral replication in IRF-1^−/−^ mice in the late phase. Thus, in both brain parts, cerebellum and cerebrum, loss of IRF-1 leads to higher viral replication in the later phase of infection although IFN-β is expressed, indicating its non-redundant function in mediating antiviral activity.

IRF-1 is shown to have the ability to directly induce antiviral IFN stimulated genes [22]. To define the antiviral status of the brain we investigated the induction of several prominent IFN stimulated genes (ISGs) within the cerebrum where high viral replication was observed (Figure 5B). Increased RNA expression levels of the ISGs Mx2, Rsad2, and USP-18 mRNA were found in both WT and IRF-1^−/−^ 2 days post infection, followed by a decline. However, expression levels increased again on day 6 post infection in IRF-1^−/−^ mice, which correlates to IFN-β induction. IFIT-2 mRNA, which has been previously defined as an ISG to restrict VSV replication in the brain [43], was induced only at day 6 post infection. Interestingly, an increase of ISG expression in the cerebrum was also detectable in WT mice in the absence of detectable amounts of IFN-β mRNA. From this we suggest, that induction of these tested ISGs in the absence of IFN-β could be IRF-1-dependent. Immunohistological analysis showed that IFIT-2 is expressed in brains of both, WT and IRF-1^−/−^ mice, although VSV infection is not detectable in WT mice (Figure 5C). IFIT-2 expression is only detectable in non-infected cells of WT animals. In contrast, in IRF-1^−/−^ mice, VSV positive and negative cells expressed IFIT-2. Collectively, these data suggest two waves of antiviral response in the brain after i.n VSV infection. While the first wave of antiviral responses peaking at day 2 is IFN-dependent and induces long lasting ISG expression, the second wave is IRF-1-dependent and limits viral replication and spread.

### Brain specific induction of IRF-1

**Figure 6:**
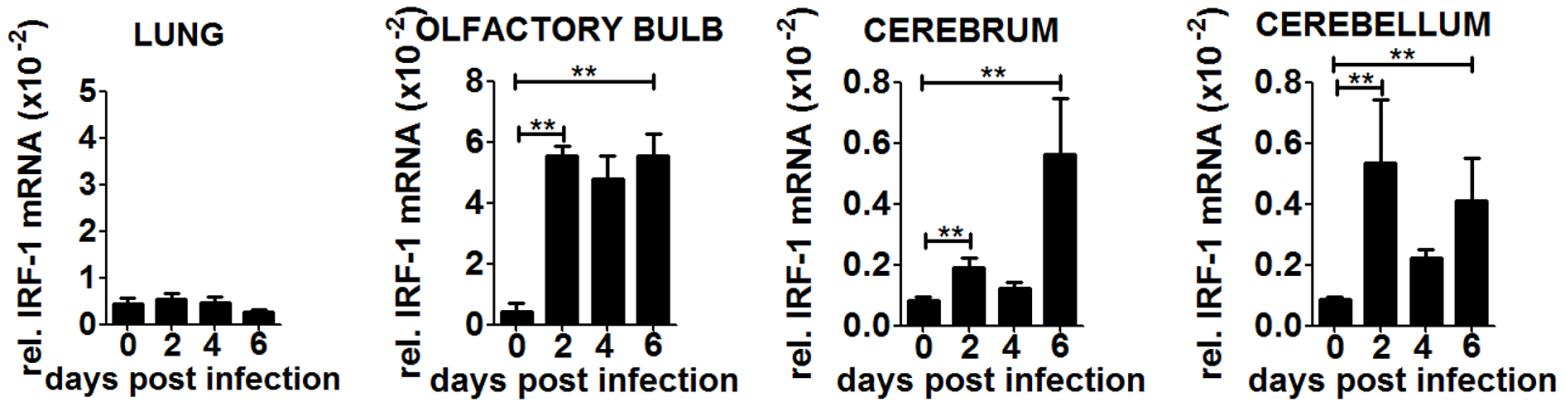
IRF-1 is induced at later states during VSV infection in the brain. WT mice were infected intranasally with 5×10^6^ pfu of VSV. Expression level of IRF-1 in the lung and different brain parts were determined by real-time RT-PCR. Asterisks indicate statistical significance calculated by Mann-Whitney test, ** p<0.005.

To confirm our hypothesis we determined IRF-1 expression in peripheral organs and the different brain parts during the course of infection (Figure 6). IRF-1 mRNA expression did not change in the lung at any time point post infection, suggesting that it does not play a role in this tissue. In contrast, IRF-1 was expressed in all the regions of the brain, albeit with different mRNA expression levels and kinetics. In the olfactory bulb, IRF-1 mRNA was induced strongly 2 days post infection and remained abundant until day 6. In the cerebrum, IRF-1 was slightly induced on day 2 but increased on 6 days post infection, whereas in the cerebellum, IRF-1 expression level peaked 2 days post infection, declined by day 4 and then was further induced by day 6. These data show that induction of IRF-1 is tissue and time point specific and could be crucial for survival against VSV induced neuropathogenesis.

To investigate if the induction of IRF-1 is dependent on IFNs, we made use of IFNAR^fl/fl^NesCre^+/−^ mice which lack IFNAR signaling in neuroectodermal cells. A strong induction of IRF-1 mRNA was detectable within different brain parts upon intranasal VSV infection (Figure S3A). However, the microglia of these mice are IFNAR competent and are by principle capable of reacting to type I IFN. Thus we cannot rule out that microglia could stimulate IRF-1 mRNA induction. However, since the IRF-1^−/−^IFNAR^fl/fl^NesCre^+/−^ mice are more susceptible (Figure S1A), this mechanism cannot not be sufficient to induce IRF-1 mediated antiviral response.

IRF-1 is known to be strongly induced by IFN-γ. Although a significant number of T cells infiltrate the brain of infected WT mice. Rag2^−/−^ mice, which lack T cells showed induction of IRF-1 mRNA in the brain (Figure S3B), indicating that infiltrating CD8+ T cells are not a source of IFN-γ which can act as an inducer of IRF-1. Since we cannot exclude that infiltrating macrophages or glia cells produce IFN-γ and thereby induce IRF-1 expression, we quantitated IFN-γ expression levels from the different brain regions. No IFN-γ mRNA induction was observed in the brains of WT mice (Figure S3C), indicating that IFN-γ is unlikely to be responsible for IRF-1 induction in the brain. Consistent with previous data, [44], IL-28 as well as the IL-28 receptor was not expressed in the brains of WT mice (Figure S3D). Collectively, these data suggest that IRF-1 expression in the brain is not induced by type I, II or III IFN. It could be induced directly by virus infection or other alternative mechanisms of PRR signaling. Importantly, both the type I IFN and the IRF-1 mediated antiviral effects are essential to restrict VSV induced neuropathogenesis.

### IRF-1mediated antiviral activity is driven by innate immune mechanisms in neurons

**Figure 7:**
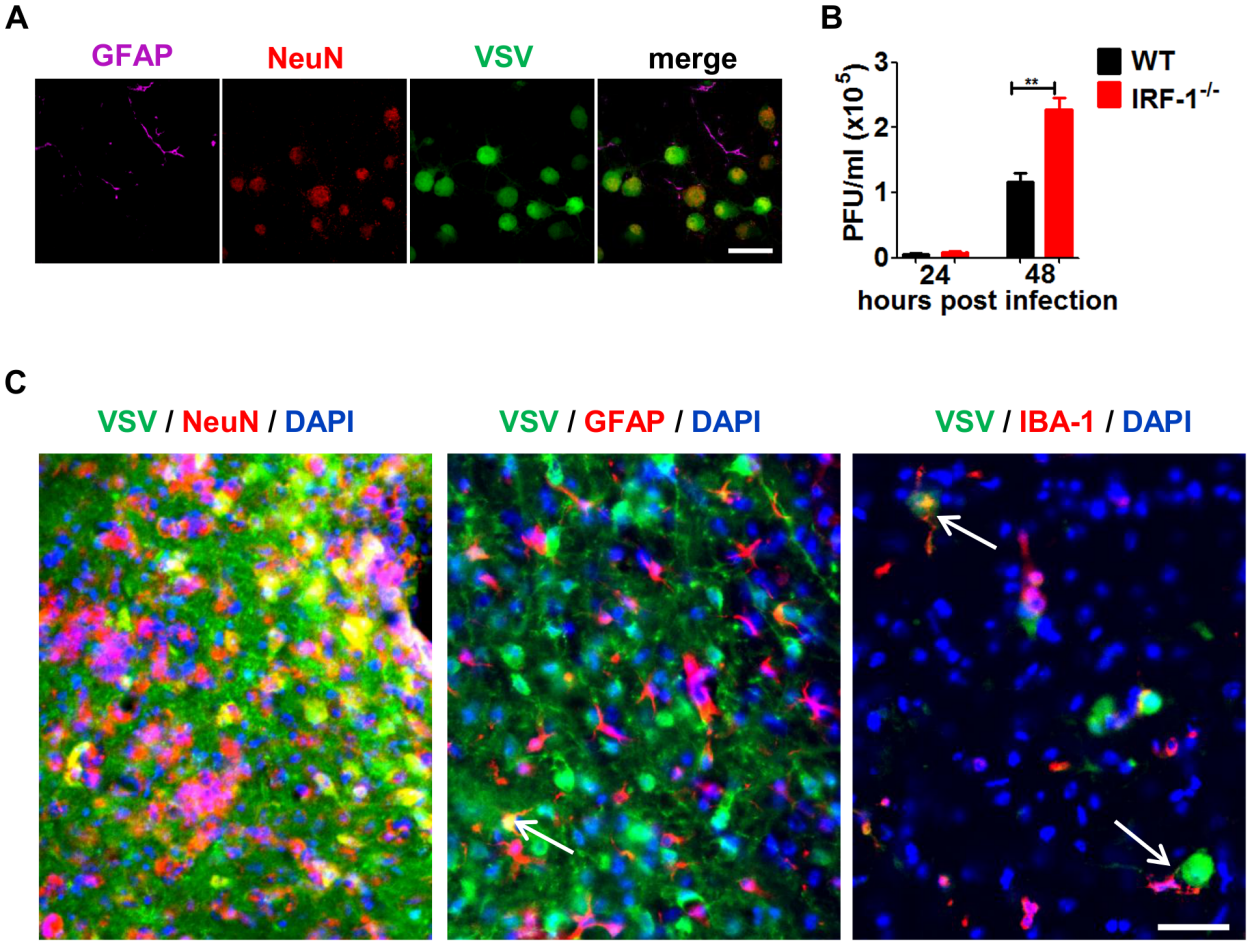
IRF-1 inhibits viral replication in primary neurons. Primary hippocampal neurons cultured from WT and IRF-1^−/−^ mice. A, Cultures were infected with 0.01 MOI VSV-eGFP and stained with GFAP (astrocytes) and NeuN (neurons) by immunofluorescence assay. Scale bar 50 μm. B, Cultures were infected with 0.001 MOI VSV and viral replication from 24–72 hours were measured by plaque assay. Asterisks indicate statistical significance calculated by Mann-Whitney test, ** p<0.005. C, IRF-1^−/−^ mice was infected intranasally with 5×10^6^ pfu VSV. Representative pictures of immunohistological analysis of VSV-eGFP (green), DAPI (blue) in neurons (NeuN: red), astrocytes (GFAP: red) of the cerebrum and microglia (IBA: red) from the brain stem 6 days post infection. Scale bar 50 μm.

The primary target for VSV replication in the brain has been reported to be the neurons [36], [43]. To assess if the source of the antiviral responses was governed by innate immune mechanisms, we monitored the viral replication kinetics in primary hippocampal cultures prepared from WT and IRF-1^−/−^ mice (Figure 7A). Using a reporter virus (VSV-eGFP), we could confirm that VSV could infect only the neurons of these cultures (Figure 7A). Neurons from IRF-1^−/−^ mice showed higher viral titers upon infection with VSV and were approximately two fold more susceptible than the WT neurons (Figure 7B). In addition, immunohistological analysis of infected IRF-1^−/−^ brains revealed an effective infection of VSV-eGFP in neurons. Within the highly infected neurons, co-staining of GFP and DAPI indicated VSV-eGFP expression in neuros. However, GFP signals in the absence of DAPI indicate VSV-eGFP expression in neuronal dendrites and axons. In contrast, the majority of astrocytes and microglia remained uninfected. Interestingly, some of the microglia was found to be close to infected cells. This shows that it is mainly the neuronal cells which are infected and possess an inherent IRF-1-dependent antiviral mechanism to defend against VSV infection.

### IRF-1 mediates antiviral responses in the brain

**Figure 8:**
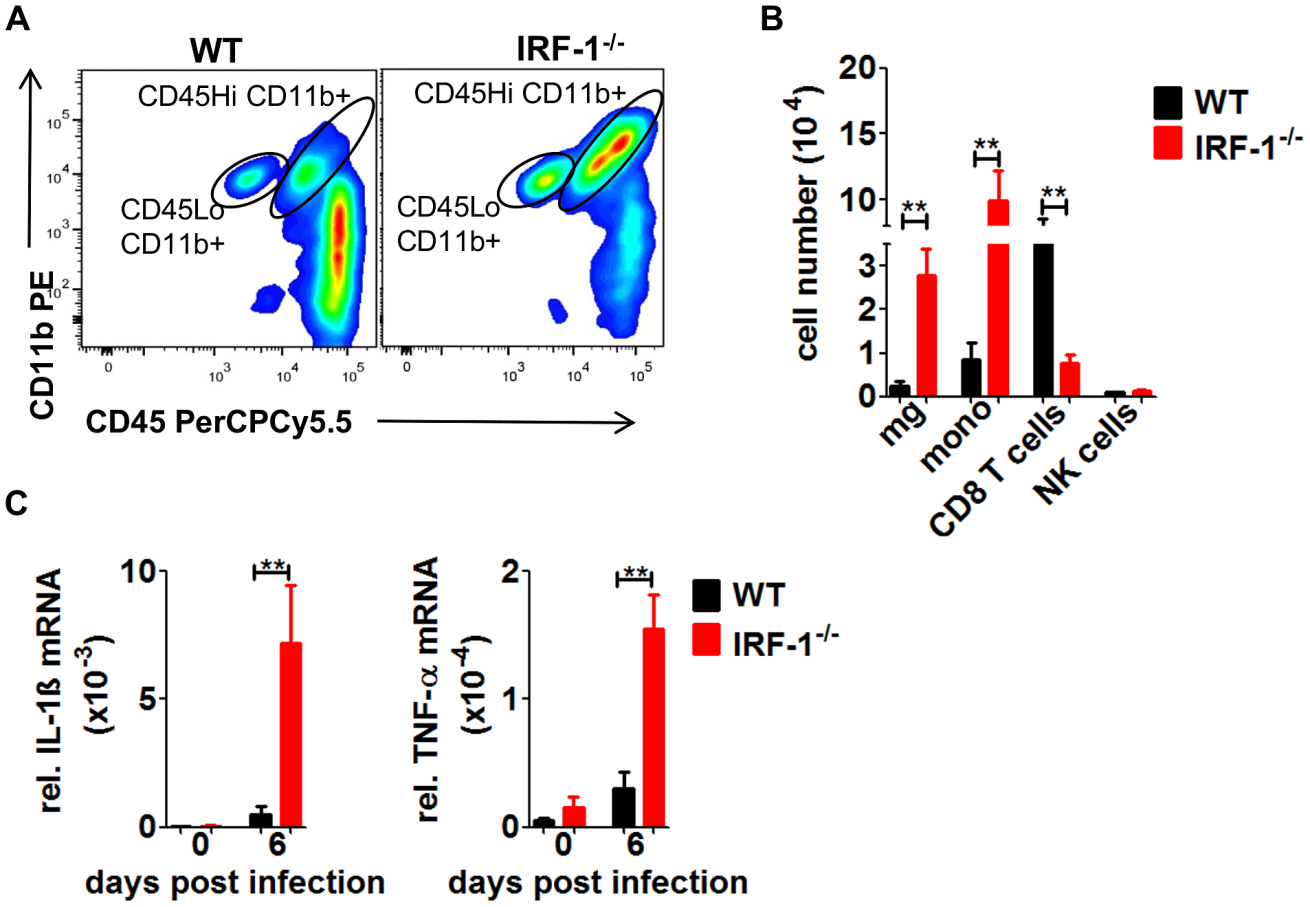
The absence of IRF-1 leads to an inflammatory response in the brain. WT and IRF-1^−/−^ mice were infected intranasally with 5×10^6^ pfu VSV. A, B, Leukocytes were isolated from brains of WT and IRF-1^−/−^ 6 days post infection by Percoll gradient centrifugation, stained for CD11b and CD45, and analyzed by flow cytometry, A, Representative flow cytometry profiles of CD11b and CD45 staining of brain leukocytes from WT and IRF-1^−/−^ mice are shown, B, Total cell numbers for infiltrating monocytes (mono) (CD45Hi CD11b+), microglia (mg) (CD45Lo CD11b+) CD8^+^ T cells (CD8^+^) and NK cells (DX5^+^CD3^−^) were evaluated. C, Expression levels of IL-1β and TNF-α mRNA were recorded by real-time RT-PCR from the cerebrum of WT and IRF-1^−/−^ mice. Asterisks indicate statistical significance calculated by Mann- Whitney test, ** p<0.005, * p<0.05.

Infected IRF-1^−/−^ mice succumb with symptoms of fatal encephalitis. To characterize the inflammatory response within brains of VSV infected mice, immune cells were isolated from brains of infected mice (Figure 8A/B). Increased numbers of infiltrating monocytes and activated microglia were detectable in IRF-1^−/−^ mice compared to their WT counterparts 6 days post infection. Neurotropic infection with West Nile virus leads to an unexpected robust CD8+ T cells response [45]. However, in VSV infection the number of CD8^+^ T cells was reduced compared to WT mice. Thus, a role of CD8^+^ T cells in the IRF-1 mediated antiviral response against VSV is unlikely (Figure 2).

To extend these findings, we measured mRNA levels of proinflammatory cytokines IL-1β and TNF-α from the cerebrum (Figure 8C). The expression of both cytokines was increased in the IRF-1^−/−^ cerebral tissues. These data suggest that higher replication of VSV by the loss IRF-1 leads to enhanced inflammatory response which could contribute to encephalitis and neurotoxicity.

## Discussion

The innate immune system is essential to limit viral replication before adaptive immunity is stimulated. The best characterized cytokine induced after virus enters a cell is the type I IFN, which subsequently mediates innate responses to inhibit viral propagation. In this study we report IRF-1 as an essential regulator of the host innate antiviral response that is not involved in the rapid IFN induction. Using IRF-1^−/−^ mice with a specific IFNAR deletion in the CNS we could demonstrate a particular IRF-1-dependent antiviral response. Detailed analysis of the viral titers in different brain regions revealed a temporal role of IRF-1 in the control of viral replication. While type I IFN plays a critical role in early stages of infection, IRF-1 is essential in limiting viral replication at later stages. Overall our results show an important role of IRF-1 in mediating a well-orchestrated antiviral response during VSV infections in the brain. We propose that IRF-1 allies with the IFN mediated responses to achieve another layer of antiviral protection to tighten the innate immune response network.

IRF-1 deficient mice show a higher susceptibility to viral infection with different viruses e.g. EMCV, murine γ-herpesvirus 68 or West Nile virus [45]–[47]. However, the antiviral response of IRF-1 is pathogen specific because some viruses can be controlled in the knockout animals [48]. Here we report that the susceptibility could be dependent on the route of infection. While IRF-1^−/−^ mice succumb to i.n. infection, all mice survive the infection by i.v. route. This could be due to the fact that this transcription factor plays a non-redundant role in certain cell types. Upon intranasal infection, VSV infects olfactory receptor neurons and is transported to the olfactory bulb via the olfactory nerves, from where the virus spreads trans-synaptically to other regions of the brain [39]. This suggests a specific effect of IRF-1 in controlling VSV infection within neurons.

Type I IFN responses orchestrate the antiviral response of many virus infections including VSV infections. Upon i.n. infection with VSV, type I IFN response acts in the glomerular layer of the olfactory bulb to prevent viral spread to the CNS [36]. The IFNAR^−/−^ mice succumbed to the infection within 3 days but the phenotype in VSV-infected IRF-1^−/−^ mice showed a different picture as IRF-1^−/−^ mice survived only until day 7, indicating that although the type I IFN system is important in antiviral control of VSV, IRF-1 is also essential to control the infection. Although IRF-1 was originally identified as an IFN inducer upon virus infection [32], [49], [50], the antiviral effect of IRF-1 was not due to a hampered type I IFN response. Instead, other members of the IRF-family have been identified as regulators for type I IFN expression [51], [52]. Analysis of viral titers in the different brain parts suggested a specific antiviral response by IRF-1. This was confirmed by infection experiments with IRF-1^−/−^NesCre^+/−^IFNAR^fl/fl^ mice which succumbed to infection significantly earlier than IRF-1^+/−^ NesCre^+/−^IFNAR^fl/fl^ mice, indicating essential antiviral responses by IRF-1 that are overlooked due to the strong effects of type I IFN. Mechanistically, IRF-1 and type I IFN are known to induce partially overlapping sets of ISGs that show broad antiviral activity against a range of viruses, but several studies have shown specific genes to be mediated directly by IRF-1 [24], [53].

Previous studies have reported the essential role of the adaptive immune response against VSV infections. Besides innate resistance mechanisms, neutralizing antibodies also control VSV replication in peripheral organs and inhibit lethal viremic spread of VSV to the CNS [54]. It has been demonstrated that IRF-1^−/−^ mice display several defects in adaptive immune response, therefore an impaired response could have contributed to death of VSV-infected IRF-1^−/−^ mice. We found normal VSV-neutralizing antibody response in the serum of IRF-1^−/−^ mice. Although T cell responses are not crucial for VSV infections as T cell deficient mice or mice with T cell depletion showed no impact on VSV replication [55], virus-specific T cells can be found. IRF-1^−/−^ mice have a blunted and dysfunctional CD8+ T cell response. However, in WNV infection CD8+ T cells were fully capable of lysing target cells and clearing viral infection from neurons and the brain [45]. In VSV infected brains, lower numbers of CD8^+^ T cells are detected. In bone marrow chimeric mice reconstituted with WT donor cells, IRF-1^−/−^ and WT recipients showed a competent T cells infiltration in the infected brains. Despite this IRF-1^−/−^ recipients showed no signs of improved survival. In addition, IFN-γ mRNA expression is low. Thus we conclude that the IRF-1 signaling in the brain resident cells is critically required to restrict virus replication.

The critical finding of this study is that the control of VSV infection in mice is carried out in two waves of antiviral responses. In the first wave, the host responds to virus entry replication and spread to the different brain parts. The response is characterized by type I IFN induction and response which is crucial to limit viral replication in WT and IRF-1^−/−^ mice. The second wave of antiviral responses is clearly IRF-1 mediated. IRF-1^−/−^ mice show a resurgence of viral load in the cerebrum, cerebellum and the brain stem. The function of IRF-1 is non-redundant to type I IFN activity and the slightly higher concentrations of IFN-β in IRF-1^−/−^ mice are possibly induced by increased viral load or immune cells activation but still succumb the infection. The strict limitation of type I IFN response to the early phase of infection could be due to its potential negative effects in CNS. Type I IFNs were recently shown to have both beneficial and detrimental effects in the central nervous system and a strict regulation is necessary to co-ordinate a tightly balanced equilibrium between cellular activation and inhibition [56]. IRF-1 could be essential to maintain that homeostasis in the brain.

VSV infection in the brain leads the virus to replicate mainly in neurons [43]. However, virus infection of astrocytes and microglia was reported from *in vitro* cultures and intranasal infection of young mice with high viral doses [57]. However, a comparison of viral load and cellular tropisms is difficult in mice of different age and different viral infection doses, because the blood brain barrier is not fully developed in 3–4 weeks old mice. We showed that in the absence of IRF-1 the majority of infected cells are neurons despite infection of some glia cells. In addition, primary hippocampal neurons isolated from IRF-1^−/−^ mice showed higher VSV replication rates compared to WT control. Recently it was reported that neurons from evolutionary distinct regions of the brain respond differently to WNV infection [58]. A similar observation was made in our model which showed differential expression of IRF-1 within the different brain regions. While no impact of IRF-1 was detectable in the olfactory bulb, all other brain regions tested shows an IRF-1-dependent decrease of viral load. This observation is also in line with VSV infection studies in primary olfactory neurons, which show no impact in IRF-1 mediated antiviral response induced by IFN-γ [59]. Our findings indicate that the increase of viral load as a consequence of a broader cellular tropism is marginal, since only a low number of microglia and astrocytes are infected. In the absence of IRF-1, VSV can more effectively spread and replicate in different regions of the brain such as the brain stem, the striatum, the internal capsule and the hippocampus which are crucial regions of the brain. The infection of the internal capsule, which connects the cerebral cortex with different parts of the thalamus and the brain stem, could explain how VSV is able to overcome natural barriers and infect additional regions in the brain.

IRF-1 can mediate antiviral effects in the absence of IFNs [60], by binding to conserved elements in the promoters of its target genes. This was also shown in STAT1^−/−^ cells in which expression of IRF-1 induces more than 100 target genes and inhibits replication of various viruses [24]. Although, it is unknown how IRF-1 is induced in detail, STAT-1 was identified as an upstream inducer of IRF-1 in VSV and KSHV *in vitro* infection models [22], [61]. From an evolutionary perspective, there is evidence pointing to IRFs being more primitive than the type I IFNs [62]. In contrast to type I IFN, IRFs are not secreted from cells indicating that an IRF-mediated antiviral response is cell intrinsic and therefore limited to infected cells. This leads us to believe that the IRF mediated antiviral defense is the ancient response while the IFN mediated action has evolved to strengthen the innate immune network.

In conclusion, antiviral response of IRF-1 was crucial for survival of viral infection. While IRF-1 did not obviously play a role in induction of type IFN, activation of adaptive immune cells and viral spread through the brain, we found that IRF-1 induces an intrinsic antiviral response, which is pivotal to control revive of viral replication at later stages of the infection. By this IRF-1 prevents induction of inflammatory response and protects mice from fatal brain inflammation. Given that the IRF family is crucial for host defense immunity against pathogens, a more detailed understanding of how the IRF system's signaling pathways are turned on and off could make the IRF family an attractive target for therapy for infectious diseases.

## Materials and Methods

### Ethics statement

All animal experiments were performed in compliance with the German animal welfare law (TierSchG BGBl. S. 1105; 25.05.1998). The mice were housed and handled in accordance with good animal practice as defined by FELASA. All animal experiments were approved by the responsible state office (Lower Saxony State Office of Consumer Protection and Food Safety) under permit number AZ 33.9-42502-05-12A295 and AZ 33.14.42502-04-070/08.All animal experiments were performed according to the guidelines of the German Animal Welfare Law (AZ 33.9-42502-05-12A295 and AZ 33.14.42502-04-070/08).

### Mice and infections

C57BL/6 (WT) mice were purchased from Harlan. IRF-1^−/−^, Mx2Luc, IFNAR^fl/fl^NesCre^+/−^ mice on C57BL/6 background were bred under specific pathogen-free conditions at the Helmholtz Centre for Infection Research. VSV and VSV-eGFP were propagated in Vero B4 cells and Influenza A virus PR8 in Madin-Darby canine kidney (MDCK) cells. During intranasal infections (i.n), 8-12-week-old mice were first anesthetized by i.p. injection of a mixture of ketamine (100 μg/g body weight) and xylazine (5 μg/g body weight) and then were infected with 5×10^6^ plaque forming units (pfu) of VSV in 20 μl PBS unless otherwise indicated. Mice which lost more than 20% of their body weight were sacrificed.

### Virus quantification from tissues

Virus titers from tissues were carried out as previously described with minor modifications [36]. In brief, VSV infected mice were euthanized and blood was removed from organs by cardiac perfusion with 20 ml of PBS. Organs were snap-frozen in liquid nitrogen, weighed; homogenized using Fast Prep 24 (MP) in 1.5 ml of PBS and virus was titered in 10-fold serial dilutions on Vero cells by plaque assay. Results are expressed as plaque-forming units (pfu) per gram of tissue.

### IFN-α ELISA

The amount of murine IFN-αs in mouse serum was determined by enzyme-linked immunosorbent assay (ELISA) according to the manufacturer's instructions (eBioscience).

### In vivo imaging

For *in vivo* imaging, mice were injected intravenously with 150 mg/kg of D-luciferin in PBS (CaliperLS), anesthetized using Isoflurane (Baxter) and monitored using an IVIS 200 imaging system (CaliperLS). Photon flux was quantified using the Living Image 3.2 software (CaliperLS).

### Virus neutralisation assay

Quantification of VSV specific IgM and IgG responses were done as described previously [63].

### Bone marrow chimeras

Bone marrow chimeras were generated with modifications to the previously described protocol [64]. In brief, 6–8 week old WT and IRF-1^−/−^ mice were irradiated with 950 rad and were i.v. reconstituted with 1 ×10^7^ bone marrow cells isolated from the tibia and femur of 4–6 week old IRF-1^−/−^ or WT (CD45.1) mice. Six-eight weeks after bone marrow transplantation, WT (CD45.1)→WT (CD45.2), IRF-1^−/−^ (CD45.2)→WT (CD45.1), WT (CD45.1)→IRF-1^−/−^ were bled to confirm chimeras and then infected with VSV (5×10^6^ pfu i.n). Survival of chimeras was monitored.

### Quantification of brain leukocytes

Brain leukocytes were quantitated from a protocol previously described [65]. In brief, whole brains were harvested from uninfected or i.n VSV infected mice. Following perfusion, brains were homogenized through a 70 μm cell strainer, digested with a collagenase solution (500 μg/ml collagenase D, 0.1 μg/ml trypsin inhibitor TLCK, 10 μg/ml DNase I, 10 mM HEPES in HBSS) for 1 h at room temperature. Cells were separated by centrifugation on a discontinuous 70-to-30% Percoll gradient. For detection of resident microglia and infiltrating macrophages, cells were incubated with anti-CD45 and anti-CD11b antibodies (BD Biosciences). Infiltrating T cells were detected using anti-CD3, anti-CD4 and anti- CD8 antibodies (BD Biosciences). Brain leukocyte numbers were quantitated using TruCount beads (BD Biosciences).

### RNA extraction and real-time RT-PCR

Mice organs were homogenized in Trizol reagent (Invitrogen) using tissue homogenizer FastPrep-24 (MP). 1–5 μg of total RNA was used for cDNA synthesis using First Strand cDNA synthesis (GE). qPCRs were carried out in 96 well-format real-time PCRs in a Roche LightCycler 480 II using Applied Biosystem's SYBR Green PCR core reagents. Murine PCR primers for β-actin (forward primer, 5′-TGG AAT CCT GTG GCA TCC ATG AAA-3′ and reverse primer, 5′- TAA AAC GCA GCT CAG TAA CAG TCC G-3′), IFN-β (forward primer, 5′- CTTCTCCGTCATCTCCATAGGG-3′ and reverse primer, 5′- CACAGCCCTCTCCATCAACT-3′), IRF-1 (forward primer, 5′- CTC ACC AGG AAC CAG AGG AA-3′ and reverse primer, 5′- TGA GTG GTG TAA CTG CTG TGG-3′), Mx2 (forward primer, 5′- TCA CCA GAG TGC AAG TGA GG-3′ and reverse primer, 5′-CAT TCT CCC TCT GCC ACA TT-3′), Rsad2 (forward primer, 5′- GTC CTG TTT GGT GCC TGA AT-3′ and reverse primer, 5′- GCC ACG CTT CAG AAA CAT CT-3′), USP-18 (forward primer, 5′- AAG GAC CAG ATC ACG GAC AC-3′ and reverse primer, 5′- CAT CCT CCA GGG TTT TCA GA-3′), IFIT-2 (forward primer, 5′- CAC CTT CGG TAT GGC AAC TT-3′ and reverse primer, 5′- GCA AGG CCT CAG AAT CAG AC-3′), IFN-γ (forward primer, 5′- TGG CTC TGC AGG ATT TTC ATG- 3′ and reverse primer, 5′-TCA AGT GGC ATA GAT GTG GAA GAA-3′), IL28R (forward primer 5′- CCC TGT TTC CTG ACA CTC CC-3′, reverse primer 5′- TCA GAA AAG TCC AGT GCC CG-3′), IL-28 (forward primer 5′- AGC TGC AGG CCT TCA AAA AG-3′, reverse primer 5′- TGG GAG TGA TTG TGG CTC AG-3′), IL-1β (forward primer, 5′- TCA TTG TGG CTG TGG AGA AG-3′ and reverse primer, 5′-TAA TGG GAA CGT CAC ACA CC-3′), TNF-α (forward primer, 5′- TGG GAG TAG ACA AGG TAC AAC CC-3′ and reverse primer, 5′-CAT CTT CTC AAA ATT CGA CTG ACA A-3′).

### Neuronal cell isolation and infection

Primary cultures of mouse hippocampal neurons were prepared from mouse embryos E18. Embryos were decapitated and the brains were kept in ice-cold Gey's balanced salt solution supplemented with glucose. After dissection the hippocampi were dissociated by 30 min incubation with trypsin followed by mechanical separation using a Pasteur pipette. 70.000 Cells were seeded on poly-L-lysine coated cover slips and incubated in Neurobasal medium (Invitrogen) supplemented with 2% B27 (Invitrogen), 1 x N-2 supplement (Invitrogen) and 0.5 mM Glutamax at 37°C, 5% CO2 and 99% humidity. After 3 weeks, cells were infected with either VSV (MOI-0.001). Kinetics of viral replication was recorded by plaque assay.

### Immunofluorescence assay

Cultured neuronal cells infected with VSV-eGFP (MOI-0.01) were fixed with 4% (w/v) paraformaldehyde (PFA) in in 0.1 M phosphate buffer for 20 min, and incubated in blocking solution consisting of 0.2% (v/v) Triton X-100, 1% (w/v) bovine serum albumin (Applichem) and 10% goat serum in PBS. Primary antibody incubation was performed overnight in blocking solution without BSA using markers for neurons-(antiNeuN, Millipore, 1∶500) and astrocytes (anti-GFAP (Synaptic Systems, 1∶500). Secondary antibodies conjugated with Cy3 or Cy5 (Jackson Immunoresearch) were incubated at room temperature for 2 hours diluted 1∶500 in PBS. Images were acquired on LSM Carl Zeiss Confocal.

### Immunhistology

Immunhistological analysis were performed from WT and IRF-1^−/−^ mice infected with VSV-eGFP at different time points (n = 3 per time point). Brains were removed after cardiac perfusion with PBS followed by 4% PFA and incubated 24 hours in 4% PFA followed by an incubation in 30% sucrose in 0.1 M phosphate buffer for an additional 24 hours. Subsequently, the brains were frozen in Tissue Tek Compound at −80°C. 30 μm sagittal slices of the whole brain were cut using a freezing microtome (Frigomobil, Leica, Germany). All staining procedures were performed free floating. Following a 1 hour blocking step at room temperature in PBS containing 1% BSA, 0.2% Triton and 10% goat serum the slices were incubated over night at 4°C in primary antibody solution consisting of 10% goat serum and 0.2% Triton in PBS. The following antibodies were used: monoclonal mouse anti-GFAP (Sigma, 1∶500), monoclonal mouse anti-GFP (Millipore, dilution 1∶800) polyclonal rabbit anti-GFP (Millipore, dilution 1∶2000), monoclonal mouse anti-NeuN (Milipore, 1∶500), polyclonal rabbit anti-IBA1 (Synaptic System, 1∶500). Secondary anti-mouse or anti-rabbit antibodies conjugated with Cy2, Cy3, or Cy5 (Jackson ImmunoResearch) were incubated 1∶500 in PBS for 2 h at room temperature. All analyzed samples were comparable and support the conclusion. Representative pictures are shown.

## Supporting Information

Figure S1 **IRF-1 plays little role in defense against influenza A virus infection.** A, Survival curves of WT and IRF-1^−/−^ mice after intranasal infection with 0.04 MLD50 Influenza A virus PR8/A/34 (n = 7). Data are representative of at least two independent experiments. B, Survival analysis of IRF-1 sufficient (IRF-1^−/−^) (n = 3) or deficient (IRF-1^−/−^) (n = 5) IFNAR^fl/fl^NesCre^+/−^ mice after intranasal infection with 5×10^6^ pfu of VSV. Survival differences were tested for statistical significance by the log-rank.
(TIF)

Figure S2 **Type I IFN response in the IRF-1^−/−^ mice are not compromised.** Luciferase activity from the homogenized, liver, spleen, lung and the brain of uninfected or infected IRF-1^+/−^Mx2luc and IRF-1^−/−^Mx2luc transgenic mice were quantitated at the indicated days post infection (d.p.i). Data represents mean with SEM (n = 4–8).
(TIF)

Figure S3 **IRF-1 mediates IFN-independent antiviral response**. WT, IRF-1^−/−^, IFNAR^fl/fl^NesCre^+/−^ and Rag2^−/−^ were infected intranasally with 5×10^6^ pfu VSV. Relative mRNA expression levels in the brains were determined by real-time RT-PCR at the indicated time points post infection. A/B, mRNA expression of IRF-1 in IFNAR^fl/fl^NesCre^+/−^ mice at 5 days post infection (A) or Rag2^−/−^ at six days post infection (B). C, mRNA expression of IFN-γ in WT and IRF-1^−/−^ mice. D, mRNA expression of IL28 and IL28R in cerebrum of WT mice. Positive controls included lung samples of VSV infected WT mice from day 4 post infection.
(TIF)
