# Supplementary figures and images for: Interferon Regulatory Factor-1 Protects from Fatal Neurotropic Infection with Vesicular Stomatitis Virus by Specific Inhibition of Viral Replication in Neurons
Source: PLoS Pathog. 2014 Mar 27;10(3):e1003999. doi: 10.1371/journal.ppat.1003999 (PMC3968136; doi:10.1371/journal.ppat.1003999)

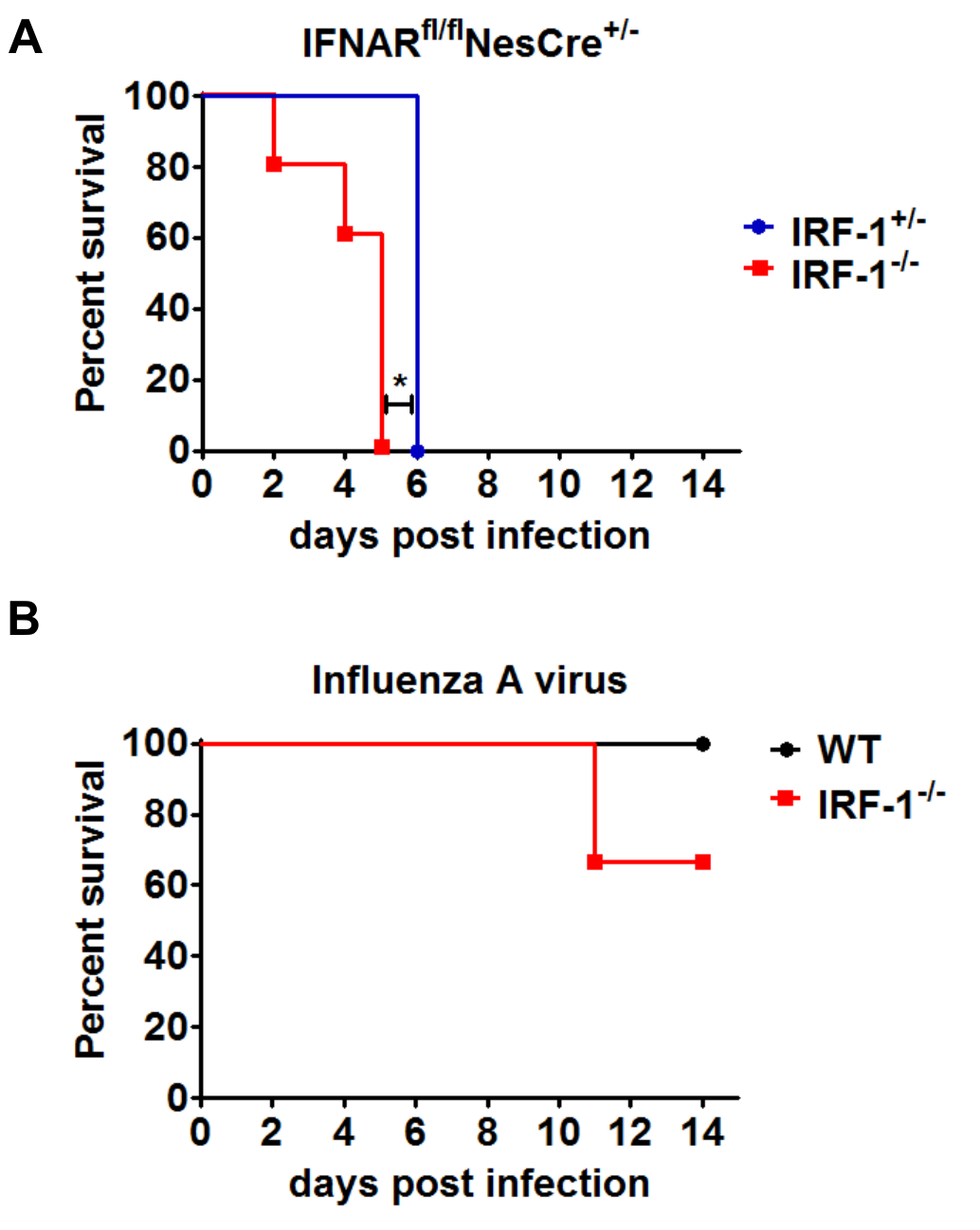

Supplement: Figure S1 — IRF-1 plays little role in defense against influenza A virus infection. A, Survival curves of WT and IRF-1−/− mice after intranasal infection with 0.04 MLD50 Influenza A virus PR8/A/34 (n = 7). Data are representative of at least two independent experiments. B, Survival analysis of IRF-1 sufficient (IRF-1−/−) (n = 3) or deficient (IRF-1−/−) (n = 5) IFNARfl/flNesCre+/− mice after intranasal infection with 5×10^6^ pfu of VSV. Survival differences were tested for statistical significance by the log-rank. (TIF) [file ppat.1003999.s001.tif]

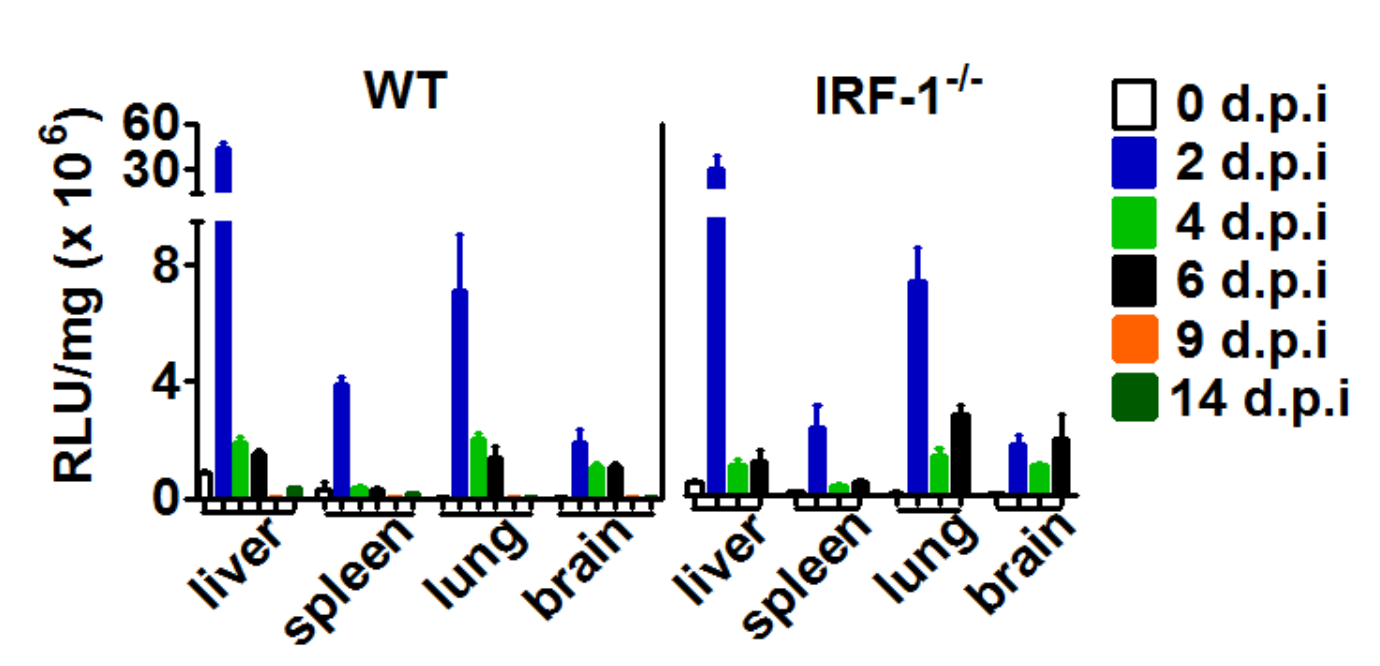

Supplement: Figure S2 — Type I IFN response in the IRF-1−/− mice are not compromised. Luciferase activity from the homogenized, liver, spleen, lung and the brain of uninfected or infected IRF-1+/−Mx2luc and IRF-1−/−Mx2luc transgenic mice were quantitated at the indicated days post infection (d.p.i). Data represents mean with SEM (n = 4–8). (TIF) [file ppat.1003999.s002.tif]

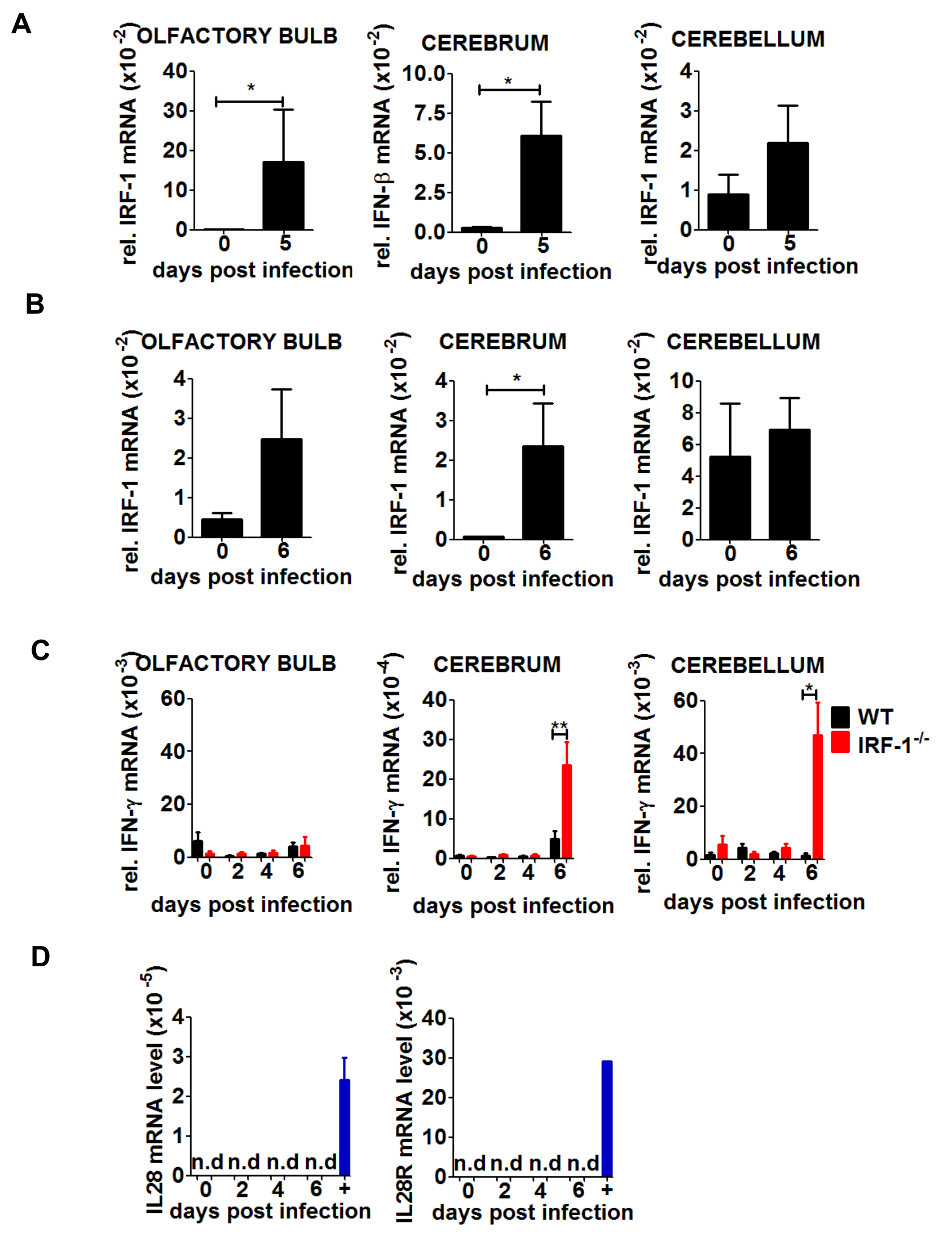

Supplement: Figure S3 — IRF-1 mediates IFN-independent antiviral response. WT, IRF-1−/−, IFNARfl/flNesCre+/− and Rag2−/− were infected intranasally with 5×10^6^ pfu VSV. Relative mRNA expression levels in the brains were determined by real-time RT-PCR at the indicated time points post infection. A/B, mRNA expression of IRF-1 in IFNARfl/flNesCre+/− mice at 5 days post infection (A) or Rag2−/− at six days post infection (B). C, mRNA expression of IFN-γ in WT and IRF-1−/− mice. D, mRNA expression of IL28 and IL28R in cerebrum of WT mice. Positive controls included lung samples of VSV infected WT mice from day 4 post infection. (TIF) [file ppat.1003999.s003.tif]
